# Fast Ripples as a Biomarker of Epileptogenic Tuber in Tuberous Sclerosis Complex Patients Using Stereo-Electroencephalograph

**DOI:** 10.3389/fnhum.2021.680295

**Published:** 2021-06-16

**Authors:** Yangshuo Wang, Liu Yuan, Shaohui Zhang, Shuangshuang Liang, Xiaoman Yu, Tinghong Liu, Xiaofeng Yang, Shuli Liang

**Affiliations:** ^1^Department of Functional Neurosurgery, Beijing Children’s Hospital, Capital Medical University, Beijing, China; ^2^Department of Neurosurgery, Fourth Medical Center, General Hospital of PLA, Beijing, China; ^3^Bioland Laboratory, Guangzhou, China

**Keywords:** epilepsy surgery, epileptogenic zone, fast ripples, stereo-electroencephalography(SEEG), tuberous sclerosis complex

## Abstract

**Objectives:** To evaluate the value of fast ripples (FRs) (200–500 Hz) recorded with stereo-electroencephalograph (SEEG) in the localization of epileptogenic tubers in patients with tuberous sclerosis complex (TSC).

**Methods:** Seventeen TSC patients who underwent preoperative SEEG examination and resective epilepsy surgery were retrospectively enrolled. They were divided into two groups according to the seizure control at 1-year postoperative follow-up. The occurrence frequencies of FRs were automatically counted, and the FR rate was calculated. The high FR rate was defined as FR rate ≧0.5. According to different positions, the contacts’ locations were divided into three groups: inner of the tubers, the junction region of the tubers, and out of the tubers. The influence factors of postoperative seizure freedom were also analyzed.

**Results:** Twelve patients reached postoperative seizure freedom at 1-year follow-up. In total, FRs were found in 24.2% of the contacts and 67.1% of the tubers in all assessed patients. There were 47 high FR rate contacts localized in the junction region of the tubers, which was 62.7% of the 75 high FR rate contacts in total and was 8.4% of the total 561 contacts localized in the junction region of the tubers. Total removal of epileptogenic tubers and total resection of the high FR rate tubers/contacts were associated with postoperative seizure freedom (*P* < 0.05).

**Conclusion:** FRs could be extensively detected in TSC patients using SEEG, and high FR rate contacts were mostly localized in the junction region of the epileptogenic tuber, which could aid in the localization of epileptogenic tubers.

## Highlights

- Fast ripples can be detected extensively by stereo-electroencephalograph in tuberous sclerosis complex patients.
- Fast ripples with high rates of occurrence frequency can be used to localize epileptogenic tubers.
- Fast ripples with high rates of occurrence frequency are mostly detected in the junction region of the epileptogenic tubers.

## Introduction

Tuberous sclerosis complex (TSC) is an autosomal dominant neurocutaneous syndrome: 85% of them with *TSC1* or *TSC2* gene mutation (Curatolo and Maria, 2013; Kingswood et al., 2014; Cui et al., 2018). Epilepsy is the most common neurological comorbidity, occurring in 90% of TSC patients (Liang et al., 2017; Cui et al., 2018; Liu et al., 2020). With medication-resistant epilepsy, there are additional standard-of-care approaches for seizure control in TSC patients beyond conventional antiseizure medications, and improved intellectual development has been found to be related to longer periods of seizure remission (Liang et al., 2017). Everolimus (a rapamycin analog), ketogenic diet therapy, and vagus nerve stimulation have been reported to have a greater than 50% reduction in seizure burden in more than 70% of patients with TSC (Krueger et al., 2013; Overwater et al., 2015; Park et al., 2017). Despite those treatments, more than 50% of patients with TSC still present intractable epilepsy. Several studies have demonstrated that resective surgery is the most effective treatment for TSC patients with medication-resistant epilepsy. Furthermore, systematic reviews have shown that 56–59% of TSC patients who underwent resective surgery achieved seizure freedom, and 68–75% experienced a worthwhile reduction (>90%) in seizure frequency (Jansen et al., 2007; Fallah et al., 2013; Liang et al., 2017; Liu et al., 2020).

Nevertheless, the greatest barrier to surgical intervention in TSC is the difficulty associated with localizing epileptogenic tuber(s), as multiple and bilateral cortical tubers often occur in cases with TSC (Fallah et al., 2013; Liang et al., 2017; Yu et al., 2019). High-resolution magnetic resonance imaging (MRI), magnetoencephalography, ^11^C-positron emission tomography (PET), MRI-PET coregistration, subtraction ictal single photon emission computed tomography coregistered to MRI, and intracranial electroencephalography (EEG) have been utilized historically during preoperative assessments (Chugani et al., 2013; Kargiotis et al., 2014; Yogi et al., 2015; Sun et al., 2018; Yu et al., 2019). However, according to literatures with 10-year postoperative follow-ups, approximately 50% of TSC patients still suffered seizures following epilepsy surgery (Liang et al., 2017; Sun et al., 2018; Liu et al., 2020). To reach better postoperative seizure control, it is essential to develop newer and more efficient approaches to localize the epileptogenic tubers more accurately in TSC patients (Yu et al., 2019).

High-frequency oscillations (HFOs) (80–500 Hz) have been demonstrated to be a promising biomarker of epileptogenicity by many studies (Jacobs et al., 2008; Crépon et al., 2010; Fedele et al., 2017). The link between HFOs and the epileptogenic zone has previously been demonstrated according to clinical data indicating a correlation between the increased removal of areas with ictal HFOs and improved postsurgical outcomes (Crépon et al., 2010; Fedele et al., 2019). However, HFOs extending beyond the epileptogenic zone were reported in the majority of patients (Jacobs et al., 2010). Haegelen et al. (2013) reported that the removal of HFO-generating areas might lead to improved surgical outcomes in patients with temporal lobe epilepsy, but not in those with extratemporal lobe epilepsy. How to distinguish pathological ripples from physiological ripples is an obstacle in the clinical application of HFOs in the localization of epileptogenic zones. It is also reported that fast ripples (FRs) (200–500 Hz), but not ripples (80–200 Hz), correlate with seizure control in patients with medication-resistant epilepsy (Crépon et al., 2010; Akiyama et al., 2011; Ren et al., 2018).

In previous literatures, HFOs have been used for preoperative assessment in TSC patients with intracranial EEG recorded with subdural electrodes (Okanishi et al., 2014; Fujiwara et al., 2016). However, FRs recorded with stereo-EEG (SEEG) in TSC patients have not been studied before. Therefore, this study aims to utilize SEEG to investigate the value of FRs in localizing epileptogenic tuber(s) in TSC patients with epilepsy. We hypothesize that FRs may be a biomarker in the localization of epileptogenic tubers, and the distribution of FRs may be different in different parts of cortical tubers.

## Materials and Methods

### Patient Selection and Inclusion Criteria

Patients were enrolled following the inclusion criteria: subjects who underwent preoperative evaluations with SEEG, subjects who finished resective surgeries from January 2016 to December 2018 in our epilepsy centers in Beijing, patients who had met the criteria of medication-resistant epilepsy for no less than 2 years, and subjects who had previously been diagnosed with TSC in accordance with the revised diagnostic criteria of Northrup (Northrup and Krueger, 2013). The exclusion criteria included subjects with one to three cortical tubers; subjects with obvious lymphangioleiomyomatosis, renal angiomyolipomas, and cardiac rhabdomyomas; and patients with serious cardiac, renal, or lung dysfunction (Liang et al., 2010, 2017). This study was approved by the Ethics Committee of the Fourth Medical Center, General Hospital of PLA, and the written consent was not signed for a retrospective study.

### Non-invasive Preoperative Evaluation

Non-invasive preoperative evaluations included neurological history (e.g., clinical seizure semiology) and physical examination, MRI, long-term scalp video EEG recordings, PET, and neuropsychological testing. MRI scans included 3.0-T routine axial T1-weighted, T2-weighted, and diffusion-weighted imaging; sagittal T1-weighted imaging; and 1-mm thickness by zero interval axial and coronal T2–fluid-attenuated inversion recovery (FLAIR) imaging. The number of cortical tubers was counted using axial T2-FLAIR images. Neuropsychological tests included Wechsler Intelligence Scale IV (Chinese revision) for measuring intelligence quotient and the overall subscale of quality of life on the Quality of Life in Childhood Epilepsy Questionnaire.

### SEEG Examination and Epileptogenic Tuber Localization

Stereo-electroencephalograph electrodes with 8–16 contacts, 0.8 mm in diameter, 2 mm in length for contacts, and 1.5 mm in intercontact interval (Huake Company, Beijing, China) were embedded under generalized anesthesia for recording intracranial EEGs, in order to localize the epileptogenic tubers in TSC patients with multiple potential epileptogenic cortical tubers. The SEEG electrodes covered the potential epileptogenic cortical tubers, which had calcifications or cystic changes on MRI, had abnormal findings on PET images, or were localized in regions with focal ictal symptoms or focal ictal and/or interictal epileptiform discharges on scalp EEGs (Liang et al., 2017). In addition, the adjacent cortexes to those tubers were also covered with SEEG electrodes (Yu et al., 2019).

Data from a minimum of five habitual seizures episodes were required for further analysis and identification. The epileptogenic tuber was identified as the first tuber with initial rhythmical discharge on SEEG before clinical seizure attack. Propagating tubers were identified by secondary rhythmical discharges on SEEG before or after clinical seizure attack in 10 s after ictal EEG onset. If more than one tuber exhibited an initial rhythmical discharge on SEEG during the same seizure or during different seizure episodes, comprehensive analysis was performed by combining MRI-PET image fusion data and clinical semiology to distinguish independent epileptogenic tubers from propagating tubers (Yu et al., 2019; Liu et al., 2020).

### FR Recording and Analysis

Interictal SEEG signals were recorded with an EEG acquisition system (Natus, United States) with a sampling frequency of 0–4,000 Hz (Ren et al., 2018). Five segments of 5-min interictal SEEGs during slow-wave sleep at midnight were used to analyze the occurrence frequency of FRs. Those segments were separated from each other and from seizure episodes by a minimum of 2 h. Slow-wave sleep was defined by the presence of more than 25% delta activity in 30-s epochs by visual inspection (Urrestarazu et al., 2007).

Bipolar montage with pairs of two adjacent EEG electrodes successively connected was used. The reference electrodes were excluded from the dataset. The automated detection of FRs was performed using software and previously described methods (Ren et al., 2018). During analysis, each contact and tuber with FRs were counted for every epoch and then averaged for a 5-min interval. The occurrence frequency of FRs was described with the number of FRs and the rate of FRs in each contact. The FR rate was calculated using the following formula: (the number of FRs in this contact/the maximum number of FRs among all contacts of this patient). A contact was defined as an FR contact when the occurrence frequency of FRs was more than 0.2/min in this contact. Similarly, a tuber with no less than one FR contact was defined as an FR tuber. In addition, when the FR rate was more than 0.5 in a certain contact (Burnos et al., 2014), we defined it as a high FR rate contact. Also, if no less than one high FR rate contact occurred in a tuber, we defined the tuber as a high FR rate tuber.

**FIGURE 1:**
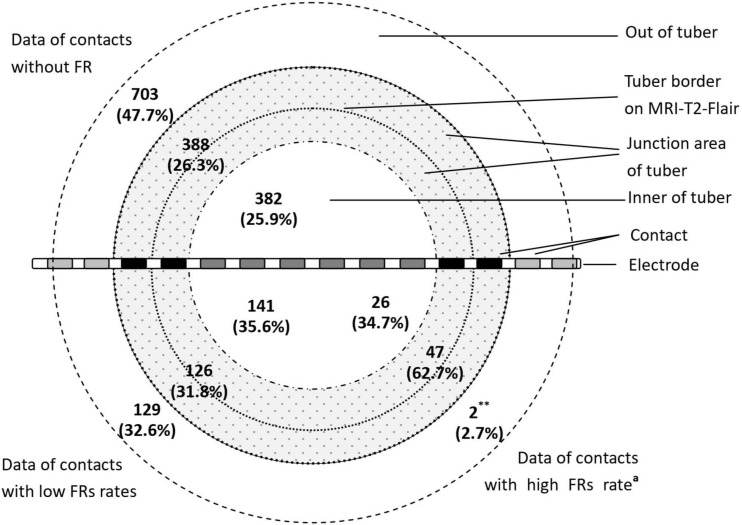
The distribution of contacts with FRs in different parts of tubers (^∗∗^*P* < 0.01, the data in this group compared with the data of contacts without FRs and data of contacts with low FR rates. ^*a*^The ratio of the high FR rate was more than 0.5). The figure shows distribution of FRs across different parts of the tuber. The majority of the high FR rate (ratio of occurrence frequency of fast ripples ≧0.5) presented in the junction area of the tubers.

According to the positional relation of contacts and tubers, the locations of contacts were defined as inner of the tuber, junction region of the tuber, and out of the tuber. The inner of the tuber was defined as inner three-fourths of the tuber. The junction region of the tuber was defined as the outer quarter area of the tuber border on MRI-T2-FLAIR plus the adjacent cortical area of gyrus with tuber involvement. *Out of the tuber* means the cortex of gyrus without tuber involvement (Figure 1).

### Surgical Approaches and Postoperative Medical Treatments

Patients underwent either lobectomies or tuber resections. Tuber resections were used for epileptogenic tubers within or close to eloquent areas. Lobectomies were applied when epileptogenic tubers were in the anterior temporal lobe or the frontal pole. Multiple tuber resections or lobectomies with tuber resection were considered in cases of multiple epileptogenic tubers unable to be removed by a single lobectomy. Pharmaceutical treatments were provided to all postoperative patients, utilizing optimized combinations of two to four kinds of antiseizure medications. Potential medications used in postoperative patient care were topiramate, vigabatrin, valproate, levetiracetam, lamotrigine, and oxcarbazepine.

### Statistical Analysis

Statistical analysis was completed using the SPSS statistical program (version 19.0; SPSS, Inc., Chicago, IL, United States). Postoperative seizure controls were classified according to the Engel method into class I (seizure free), class II (rare seizures), class III (>90% reduction in seizure frequency), and class IV (<90 reduction in seizure frequency). Outcomes were described with percentages, means, and SD. Univariate analysis of categorical variables was performed using χ^2^ and Fisher exact tests. *t*-tests and *F*-tests were used for comparison of continuous variables. When the two-tailed error probability *P* was less than 0.05, the outcome was considered to be significant.

## Results

### Patients and Presurgical Evaluations

A total of 46 patients with TSC underwent epilepsy surgery at our hospitals from January 2016 to December 2018. Twenty-one patients received implanted SEEG electrodes and underwent resective surgery, and 17 of them with comprehensive FRs data were included in this study (Table 1). Both female (*n* = 6) and male (*n* = 11) patients were presented. Patient ages ranged from 2.9 to 12.6 (mean = 6.13 ± 3.06) years. Types of clinical seizures at onset included generalized epileptic spasms (*n* = 9), generalized tonic–clonic seizure (*n* = 3), focal seizure (*n* = 4), and generalized clonic seizure (*n* = 1). Seizure frequencies included either daily seizures (*n* = 14) or weekly seizures (*n* = 3). Age at seizure onset ranged from 0.2 to 5.9 (mean = 1.34 ± 1.60) years. The durations of preoperative seizures ranged from 2.3 to 9.6 (mean = 4.79 ± 2.37) years. Through observation and counting, each patient had 10.18 ± 3.21 (range = 4–16) cortical tubers.

**TABLE 1:** Patients’ clinical and demographic characteristics and FR data.

**No.**	**Gender**	**Age at operation (years)**	**Age at seizure onset (years)**	**Seizure type**	**Drugs**	**Number of tubers**	**Number of electrodes**	**Number of tubers covered with SEEG**	**Total number of contacts**	**Contacts containing FRs (%)**	**Number of FRs in contacts containing FRs (mean ± SD)**	**Tubers with FRs (%)**	**Onset tuber**	**Propagating tuber**	**Resected tubers**	**Follow-up (years)**	**Seizure free at 1 year**
1	Male	8.2	5.9	GCTS CPS	VPA/LMT	4	4	4	52	8 (15.4)	125.63 ± 122.39	3 (75.0)	R-T	R-P R-F	R-T R-F	3.7	Yes
2	Female	4	0.2	CPS Tonic	OXC/LEV LMT/VPA	10	9	9	127	6 (4.7)	34.33 ± 37.25	3 (33.3)	L-F L-T	L-T L-O	L-T/L-F L-O	3.3	Yes
3	Male	5.8	0.8	Spasm GTCS/AA	VAP/LMT TMP	16	11	10	128	47 (36.7)	41.32 ± 74.50	10 (90.9)	R- R-F	R-FP R-TO	R-F/R-FP R-T/R-TO	2.7	Yes
4	Male	2.6	0.3	Spasm CPS	VPA/LMT LEV/RPM	8	7	8	104	17 (16.3)	9.35 ± 19.36	6 (75.0)	R-P	R-T R-PC	R-P/R-PC R-T	2.6	Yes
5	Female	3.8	0.5	Spasm GTCS	VPA/LEV VGB/RPM	12	9	11	122	14 (11.5)	6.07 ± 3.67	5 (45.5)	L-F R-F	L-FP L-T L-F	L-FP L-T/L-F	2.3	Yes
6	Female	3.7	0.3	Spasm CPS	VPA/LEV RPM/VGB	11	9	11	126	3 (2.4)	3.33 ± 2.31	2 (18.2)	R-P	R-I R-T	R-T/R-P R-I	1.8	Yes
7	Male	4.3	0.3	Spasm tonic	VPA/CLB RPM/VGB	14	10	12	121	26 (21.5)	28.50 ± 55.07	7 (58.3)	R-T	R-F	R-T R-F	1.7	Yes
8	Male	8.1	4	CPS GTCS	VPA/LMT RPM	9	10	9	124	33 (26.6)	55.61 ± 80.61	8 (88.9)	L-P	L-T L-PC	L-P/L-PC L-T	1.6	Yes
9	Male	3.8	0.8	Clonic/AA GCTS	VPA/TMP LEV	8	8	8	116	28 (24.1)	32.61 ± 21.86	7 (87.5)	R-F R-T	R-I R-FC	R-F/R-FC R-T/R-I	1.3	Yes
10	Male	7	0.3	Spasm GCTS	LEV/TMP VGB/LMT	11	9	10	127	24 (18.9)	157.42 ± 171.45	4 (40)	R-F	L-F R-T	R-F/L-F	1.2	Yes
11	Male	6.6	1	CPS/tonic GTCS	VPA/CBZ VGB/RPM	11	9	9	126	34 (27.0)	100.09 ± 170.57	8 (88.9)	L-F	L-P L-T	L-F/L-P L-T	1.1	Yes
12	Male	11	2	CPS GTCS	CBZ/VPA LMT	6	6	6	90	43 (47.8)	108.52 ± 218.28	6 (100)	L-P	L-F L-I	L-P/L-F L-I	1.1	Yes
13	Female	4.8	0.4	Spasm CPS	OXC/VPA VGB/RPM	7	7	7	104	51 (49.4)	77.16 ± 73.16	7 (100)	R-F L-F	R-T/R-P R-FC	R-F/R-T R-P/R-FC	2.9	No
14	Female	10.8	2	GTSC myoclonic	LEV/LMT VPA/RPM	14	8	10	116	4 (3.4)	4.75 ± 3.59	3 (30)	L-T	L-O/L-P	L-T/L-O L-P	2.8	No
15	Male	4.2	0.4	Spasm tonic AA	VPA/OXC LMT/VGB RPM	8	9	8	124	18 (14.5)	50.61 ± 41.78	6 (75.0)	R-Central R-F	R-F/L-F R-C	R-F R-Central R-C	1.8	No
16	Female	12.6	3	GCTS CPS	LEV/VGB VPA	10	9	9	110	53 (48.2)	25.45 ± 38.33	7 (77.8)	R-F L-P	R-T/R-I R-TO	R-F/R-I R-T R-TO	1.7	No
17	Male	2.9	0.5	Spasm tonic	LEV/CLB RPM/VGB	14	10	11	127	62 (48.8)	11.63 ± 22.39	10 (90.9)	L-F R-P	L-I/L-C	L-F/L-I L-C	1	No

*AA, atypical absence; C, cingulate gyrus; CPS, complex partial seizure; F, frontal lobe; GTCS, generalized tonic-clonic seizure; I, insular lobe; L, left; NA, no available; O, occipital lobe; P, parietal lobe; R, right; T, temporal lobe; CBZ, carbamazepine; CLB, clobazam; LEV, levetiracetam; LMT, lamotrigine; OXC, oxcarbazepine; RPM, rapamycin; TMP, topiramate; VGB, vigabatrin; VPA, valproate.*

### Surgical Approaches and Outcomes

A total of 25 epileptogenic tubers were identified across all patients. Patients were observed to have a single epileptogenic tuber (*n* = 9) or two epileptogenic tubers (*n* = 8); no patient was observed to have more than two epileptogenic tubers. Furthermore, 35 early propagating tubers were identified. Surgical interventions varied case by case and included epileptogenic tuber resection (*n* = 6), lobectomy or multilobar resection (*n* = 7), and a combination of lobectomy and tuber resection (*n* = 4). In total, 52 tubers, including 22 epileptogenic tubers and 30 propagating tubers, were removed. At 1-year follow-up, 12 patients (70.6%) achieved seizure freedom (Engel I), one reached Engel II, and the other four cases reached Engel III–IV seizure control. Significant difference was found in the percentage of total removal of epileptogenic tubers between patients with postoperative seizure freedom and those with postoperative continuous seizure at 1-year follow-up (*P* = 0.0239) (Table 2).

**TABLE 2:** Influence factors of postoperative seizure freedom.

**Factors**	**Patients with seizure freedom (*n* = 12)**	**Patients with continuous seizure (*n* = 5)**	***P*-value**
Age at surgery (years)	5.53 + 2.49	7.06 + 4.34	0.3675
History of seizure (years)	4.63 + 2.21	5.80 + 3.20	0.3954
Age at first seizure (years)	1.37 + 1.79	1.26 + 1.19	0.9021
Patients with epileptic spasm (count/%)	6/50.0%	3/60.0%	0.8754
Patients with partial seizure (count/%)	7/58.3%	2/40.0%	0.7066
Number of cortical tubers	9.50 + 3.73	10.6 + 3.27	0.5758
Number of epileptogenic tubers	1.33 + 0.49	1.80 + 0.45	0.0855
Number of propagating tubers	2.00 + 0.43	2.40 + 0.55	0.1269
Number of epileptogenic tubers and early propagating tubers	3.33 + 0.78	4.20 + 0.84	0.0580
Number of removed tubers	2.92 + 0.67	3.40 + 0.55	0.1794
Total removal of epileptogenic tubers (count/%)	12/100%	2/40%	**0.0239**
Total removal of epileptogenic and early propagating tubers (count/%)	9/75%	1/20%	0.1191
Percentage of contacts with fast ripples (%)	21.01 + 12.80	32.86 + 22.18	0.1806
Percentage of tubers with fast ripples (%)	67.55 ± 27.74	74.74 + 26.97	0.6309
Number of contacts with fast ripples	23.58 + 14.33	37.60 + 25.12	0.1609
Number of tubers with fast ripples	5.75 + 2.42	6.60 + 2.51	0.5235
Total resection of contacts with high fast ripples rate (FRs rate ≥ 0.5)	11 (91.7%)	0 (0.0%)	**0.0023**
Total resection of tubers with high fast ripples rate (FRs rate ≥ 0.5)	11 (91.7%)	0 (0.0%)	**0.0023**

*Bold values indicate the *P* < 0.05.*

### Interictal FRs and the Distribution

There were 471 (24.2%) FR contacts detected from the 1,944 contacts of 144 implanted SEEG electrodes, including 75 (3.9%) high FR rate contacts. Moreover, 102 (67.1%) FR tubers were observed across all covered tubers (*n* = 152) in all patients (Table 1).

The occurrence frequency of FR discharge significantly varied across the tuber anatomy (*P* < 0.01). There were 62.7% (47 of 75) high FR rate contacts located at the junction region of the tubers, and those contacts without FRs mainly located out of the tubers (*n* = 703, 47.7% of 1,473). High FR rate contacts included two (0.2% of 834) contacts out of the tubers, 47 (8.4% of 561) contacts in the junction region of the tubers, and 26 (4.7% of 549) contacts in the inner of the tubers (Figure 1). There were significant differences in the percentage of the high FR rate contacts in the three parts of cortical tubers (*P* = 0.0000).

### Interictal FRs and Seizure Control

The tubers and contacts with FRs were compared between patients with Engel I seizure controls and those with Engel II–IV seizure controls. There was no significant difference found in percentage (or number) of FR contacts (or tubers) between those two groups (*P* > 0.05) (Table 2).

**FIGURE 2:**
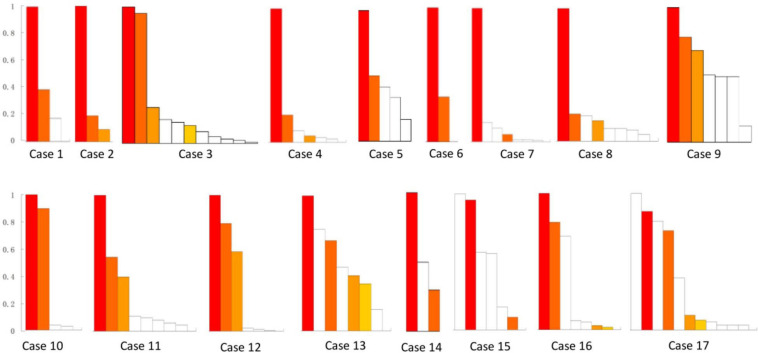
Resective range of contacts with FRs in all of the 17 patients. This figure shows the removed contacts with FRs in each patient. There were 272 FR contacts removed in total. White bars show the reserved contacts with FR discharges. Color bars show the removed contacts with FRs. The same color bars in each patient meant the removed contacts in the same electrode, whereas the different color bars in each patient meant the contacts from different electrodes. Patients 13–17 suffered continuous seizure attack after resective operations. *y-*axis was the ratio of FRs on each contact. *x-*axis was contacts in order of the ratio of FR discharges in each patient.

**FIGURE 3:**
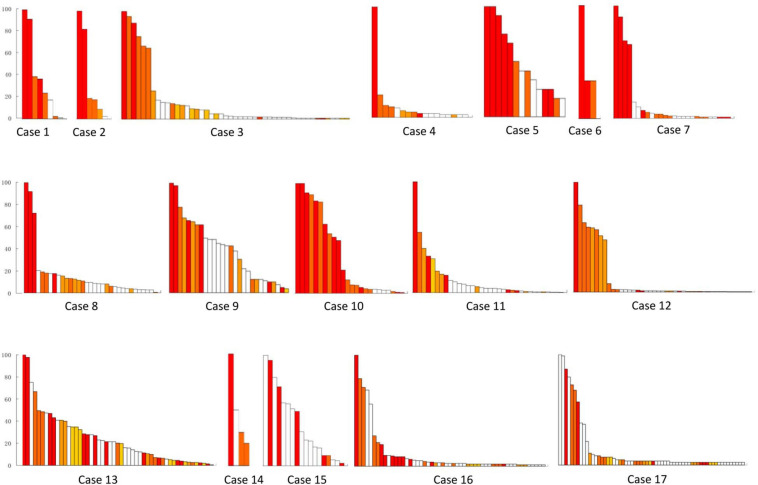
Resective range of the tubers with FRs in all of the 17 patients. This figure shows the removed fast ripple tubers in each patient, and the number was 48 in total. White bars show the reserved tubers with FR discharges. Color bars show the removed tubers with FRs, and different color bars meant different removed tubers in each patient. Patients 13–17 suffered continuous seizure attack after resective operations. *y*-axis was the ratio of FRs on each tuber. *x-*axis was tubers in order of the ratio of FRs in different tubers in each patient.

The removed brain tissues included 272 FR contacts (Figure 2) and 48 FR tubers (Figure 3) in all patients. Each patient removed 3–30 FR contacts, and the maximum rates of FRs occurrence frequency in remained contacts ranged from 0 to 1 in different cases (Figure 2). Significant differences were found in seizure freedom between patients who totally removed the high FR rate contacts and those who partially removed the high FR rate contacts (100 vs. 16.7%, *P* < 0.05) (Table 2).

Each patient removed two to four FR tubers, and the maximum FR rate in the remained tubers ranged from none to one in different cases (Figure 3). Significant differences were found in seizure freedom between those who totally removed high FR rate tubers and cases who partially removed high FR rate tubers (100 vs. 16.7%, *P* < 0.05) (Table 2).

### Complications

One patient presented asymptomatic epidural hematoma around an electrode. No complication was identified during FR recording.

## Discussion

To the best of our knowledge, this study presented the first comprehensive observations of FRs detected by SEEG in cortical tubers of TSC patients. FRs were recorded in 24.2% of SEEG contacts and 67.1% of tubers covered with SEEG electrodes. The occurrence frequency of FRs varied among patients. However, areas with FRs were relatively stable in the same patient, which was consistent with previous studies (Bagshaw et al., 2009).

Fujiwara et al. (2016) and Okanishi et al. (2014) reported the presence of ictal and interictal ripples and FRs in children with TSC recorded by subdural intracranial EEG, respectively. However, the analysis of ictal HFOs was unreliable because of various potential impacts, and the ripples could not work as a biomarker for epileptogenic onset zone (Crépon et al., 2010; Akiyama et al., 2011; Fedele et al., 2019). Fujiwara et al. (2016) found that complete resection of regions with HFOs led to a better surgical outcome. However, our study and the study by Okanishi et al. (2014) showed that extensive FRs were recorded in TSC patients, and complete resection of FR contact was uncommon, especially for patients with multiple tubers. Therefore, high FR rate contacts should be identified.

Clinicians faced multifaceted challenges related to thresholds and the identification of potential epileptogenic areas. Okanishi et al. (2014) applied a bootstrapping method for thresholding, which yielded a mean HFO rate and then used it to distinguish between high and low FR channels. Application of this method led to a significant correlation between removal of the high occurrence frequency FR channels and the postoperative seizure freedom in TSC patients with subdural electrode EEG. In this retrospective research, we used 0.5 as the cutoff ratio of high occurrence frequency of FRs to define the epileptogenic zone and tubers, which had been used by Burnos et al. (2014). Then, we found significant differences in seizure freedom between patients who totally removed contacts (tubers) with high FR rates and those who partially removed, which indicated that the threshold of 0.5 had practicability and reliability in the identification of epileptogenic tuber in TSC patients, but more data were still needed to test and verify.

Previously, the onset zones of TSC, localizing to TSC tuber itself vs. perituber cortex, were controversial (Ma et al., 2012; Kannan et al., 2016). TSC cortical tubers were observed to have dysmorphic cytomegalic and immature neurons, which played an important role in the generation and propagation of epileptic discharges (Abdijadid et al., 2015; Mühlebner et al., 2016). The perituber cortex was identified through abnormalities obtained through electrocorticography, diffusive tension image, histological pathology, immunohistochemical analysis, or molecular patterns (Oh et al., 2011; Ma et al., 2012; Krsek et al., 2013). Kannan et al. (2016) found that focal seizures and interictal epileptiform discharges raised at the center of epileptogenic tubers and propagated into the tuber rim, perituber cortex, and other epileptogenic tubers. With the use of SEEG, but not subdural intracranial EEG, the FRs in the inner of the tubers and junction area of the tubers could be recorded. In this study, we found that 62.6% of the high FR rate contacts presented on the junction region of the tuber and adjacent cortex, while the contacts with low FR rates were almost evenly distributed in the inner of the tubers, the junction area of the tubers, and out of the tubers. Therefore, the junction area of epileptogenic tubers should be the epileptogenic zones in TSC patients.

There are some limitations to this study. First, patients with one to three cortical tubers were excluded, because the epileptogenic tuber and propagative tuber need to be defined at the same time. Second, TSC patients with three or more epileptogenic tubers were not enrolled in the study, because most of them were excluded from the resective operations. Third, the sample of enrolled subjects was small because of the low incidence of TSC.

## Conclusion

In conclusion, FRs were extensively recorded in patients with TSC utilizing SEEG, and electrode contacts with high FR rates can be used to localize epileptogenic tubers. Furthermore, the junction areas of the tubers had most contacts with high FR rates and indicated the locations of epileptogenic zones.

## Data Availability Statement

The raw data supporting the conclusions of this article will be made available by the authors, without undue reservation.

## Ethics Statement

The studies involving human participants were reviewed and approved by the Ethics Committee of Fourth Medical Center, General Hospital of PLA. Written informed consent for participation was not provided because this study is a retrospective study and patients did not provide additional information or perform other treatments or examinations.

## Author Contributions

SlL and SZ performed the operative and collected candidate information. LY, ShL, and XYu finished the EEG recording. XYa, YW, LY, TL, and SlL performed HFO analyses. SlL, ShL, YW, and XYa drafted the manuscript. YW, LY, SZ, and TL analyzed the datasets. All authors contributed to the article and approved the submitted version.

## Conflict of Interest

The authors declare that the research was conducted in the absence of any commercial or financial relationships that could be construed as a potential conflict of interest. The handling editor declared a shared affiliation with several of the authors (YW, LY, SZ, TL, and SlL) at the time of review.
